# Mapping novel genetic loci associated with female liver weight variations using Collaborative Cross mice

**DOI:** 10.1002/ame2.12036

**Published:** 2018-10-24

**Authors:** Hanifa J. Abu‐Toamih Atamni, Maya Botzman, Richard Mott, Irit Gat‐Viks, Fuad A. Iraqi

**Affiliations:** ^1^ Sackler Faculty of Medicine Tel‐Aviv University Tel‐Aviv Israel; ^2^ Faculty of Life Sciences Tel‐Aviv University Tel‐Aviv Israel; ^3^ Department of Genetics University College of London London UK

**Keywords:** candidate genes, Collaborative Cross mouse model, high genetic diverse mouse population, liver weight, quantitative trait locus mapping, standard rodent diet

## Abstract

**Background:**

Liver weight is a complex trait, controlled by polygenic factors and differs within populations. Dissecting the genetic architecture underlying these variations will facilitate the search for key role candidate genes involved directly in the hepatomegaly process and indirectly involved in related diseases etiology.

**Methods:**

Liver weight of 506 mice generated from 39 different Collaborative Cross (CC) lines with both sexes at age 20 weeks old was determined using an electronic balance. Genomic DNA of the CC lines was genotyped with high‐density single nucleotide polymorphic markers.

**Results:**

Statistical analysis revealed a significant (*P* < 0.05) variation of liver weight between the CC lines, with broad sense heritability (*H* ^2^) of 0.32 and genetic coefficient of variation (CV_G_) of 0.28. Subsequently, quantitative trait locus (QTL) mapping was performed, and results showed a significant QTL only for females on chromosome 8 at genomic interval 88.61‐93.38 Mb (4.77 Mb). Three suggestive QTL were mapped at chromosomes 4, 12 and 13. The four QTL were designated as *LWL*1‐*LWL*4 referring to liver weight loci 1‐4 on chromosomes 8, 4, 12 and 13, respectively.

**Conclusion:**

To our knowledge, this report presents, for the first time, the utilization of the CC for mapping QTL associated with baseline liver weight in mice. Our findings demonstrate that liver weight is a complex trait controlled by multiple genetic factors that differ significantly between sexes.

## 1 INTRODUCTION

The liver is the second largest organ of the human body, known for complex functional anatomy involving vascular and biliary relationships, which play fundamental roles in maintenance and regulation of the body energy metabolism.^1^, ^2^ Hence, disrupted function of the liver has been found to be related to an extensive range of complex diseases and metabolic disorders.^3^, ^4^, ^5^ Hepatomegaly (liver enlargement) is a major symptom indicating malfunctioning of the liver, either due to topical disease or in response to related diseases. It is known that common diseases associated with liver enlargement may be either due to hepatocyte fatty infiltration and hepatocyte enlargement (alcoholic hepatitis and other causes of fatty liver), or to infiltration of cancer cell deposits, which are growing rapidly (metastatic cancer, lymphoma, hepatoma). Other cases of congestive hepatomegaly could be due to hepatic venous outflow obstruction (congestive heart failure).^6^, ^7^, ^8^, ^9^ Liver weight is known to be correlated with body growth, internal organ weight, metabolic trait, age and sex, which are assumed to be controlled by polygenic effects.^10^, ^11^, ^12^ Previous studies of body growth and its composition, for purposes of either medical knowledge on growth or meat‐producing industries, affirmed and significantly contributed to the understanding of the complex genetic components underlying those age‐related traits (ie, selection experiments, quantitative trait loci [QTL] mapping, genome‐wide association studies [GWAS]).^13^, ^14^, ^15^, ^16^, ^17^, ^18^, ^19^ However, the complex genetic background controlling growth in the context of liver weight is still obscure and requires the use of advanced animal models that may enable narrowing the mapped QTL intervals.

Rodent and human physiology are very similar, and various mouse models have been used widely in the study of liver anatomy and function in healthy and diseased forms, noting that animal models known so far in the study of human liver diseases manage to mimic specific features of the human disease but not all.^20^, ^21^ Given the wide genetic variation existing between human populations alongside the multiple limitations in human study (eg, weak control for standardized investigations), studying a complex human trait or disease requires a highly genetically diverse mouse population rather than a single mouse model. For this purpose, the Collaborative Cross (CC) mouse population model was designed to provide a new model population dedicated to genetic analysis of complex traits as needed for understanding complex human diseases.^22^, ^23^ This unique reference genetic resource comprises a set of approximately 350 recombinant inbred lines (RILs) created from full reciprocal matings of eight divergent strains of mice: A/J, C57BL/6J, 129S1/SvImJ, NOD/LtJ, NZO/HiLtJ, CAST/Ei, PWK/PhJ and WSB/EiJ. Aiming to create a unique and inexhaustible resource of RILs presenting a large phenotypic and genetic diversity, a controlled randomization was carried out during the breeding process to disband large linkage disequilibrium blocks and to recombine the natural genetic variation of the inbred strains.^24^ The genetic contribution of the three wild‐derived founders of the CC lines (CAST/EiJ, PWK/PhJ and WSB/EiJ) is of great importance, representing the subspecies *Mus musuclus castaneus* (*M. m. castaneus*), *M. m. musculus* and *M. m. domesticus*, respectively, which established a large number of sequence variants not segregating among classical strains.^25^ Indeed, simulation studies showed that the 100 RILs being developed at our laboratory enables mapping of a QTL explaining a total of 5% of the RIL phenotypic variation with an average genomic interval of 3.5 cM.^26^ All CC lines were genotyped using three different arrays. The Mouse Diversity Array (MDA) consisting of 620 000 single nucleotide polymorphism (SNP) markers;^27^ thereafter, all SNPs with heterozygous or missing genotypes in the eight CC founders were filtered out leaving 170 935 SNPs, which were mapped onto to build 37 of the mouse genomes. The 170 935 SNPs were clustered (groups of n* = *20 SNPs) based on their descent probability distribution, using HAPPY Hidden Markov Model (HMM) software^28^, ^29^ resulting in reduced intervals (8533), minimizing the effect of genotyping error and the analyses (faster). After the MDA, CC lines were re‐genotyped at an advanced generation with a new 7500 custom‐design SNP array, Mouse Universal Genotype Array (MUGA), providing the genome architecture of the CC lines^30^. Eventually, after five advanced generations, all CC lines were genotyped with Mega‐MUGA, and the genotypes of the three SNP arrays were merged (merge analysis) to prepare a single genotype file, which is now used successfully in QTL mapping.^31^, ^32^, ^33^, ^34^, ^35^

As of today, multidisciplinary studies using the CC lines at our laboratory and others has already demonstrated unprecedented power for high‐resolution QTL mapping (~1 Mb) by phenotyping a relatively modest number of CC lines (~30 lines) with sufficient replication.^26^, ^30^, ^31^, ^32^, ^33^, ^34^, ^35^, ^36^, ^37^, ^38^, ^39^, ^40^, ^41^, ^42^, ^43^, ^44^, ^45^, ^46^, ^47^, ^48^, ^49^

## 2 MATERIALS AND METHODS

### 2.1 Ethical statement

All experimental mice and protocols were approved by the Institutional Animal Care and Use Committee (no. M‐10‐073 and M‐14‐007) of Tel‐Aviv University (TAU), which adhered to the Israeli guidelines that follow the National Institutes of Health of USA animal care and use protocols.

### 2.2 CC lines

Full details of the development of these CC lines are given in previous reports.^50^, ^51^ The study cohort consisted of 506 mice, generated from 39 different CC lines, from which 22 CC lines were selected with representation of both sexes. Due to variations in breeding nature of the CC lines, the numbers of mice used in this study were 168 female mice generated from 24 CC lines and 338 male mice generated from 37 CC lines. Mice of the CC lines were provided at the age of 18‐20 weeks, by the Small Animal Facility at Sackler Faculty of Medicine, TAU. Mice were housed on hardwood chip bedding in open‐top cages, maintained at a 12:12‐h light:dark cycle at a temperature of 21‐23°C. The mice were given tap water and standard rodent chow diet ad libitum, which consists of %Kcal from 18% fat, 24% protein and 58% carbohydrates (TD.2018SC; Teklad Global, Harlan, Madison, WI, USA), since weaning at the age of 3 weeks until the age of 20 weeks.

### 2.3 Phenotype recording

At 20 weeks old, following 12 weeks of standard rodent diet, mice were killed by cervical dislocation after i.p. injection of anesthetic solution (ketamine/xylazine). Thereafter, mice were dissected and livers collected, and the liver weight of each mouse was determined using an electronic balance.

### 2.4 Availability of data and materials

Phenotype data presented in this study will be publically available in the Mouse Phenome Database (http://phenome.jax.org) and all SNP genotype data is available at http://mtweb.cs.ucl.ac.uk/mus/www.

### 2.5 Statistical analysis

Phenotypic variations between the CC lines were calculated by one‐way anova using the SPSS version 23 software (SPSS, Chicago, IL, USA). Significant variations were considered at *P* ≤ 0.05. Estimated heritability (*H* ^2^) and genetic coefficient of variation (CV_G_) were calculated for the phenotypic traits using the anova output (*H* ^2^ = *V*_g_ / [*V*_g_ + *V*_e_]) as shown in our previous report.^52^

### 2.6 CC line marker genotyping

Collaborative Cross lines were genotyped using three different arrays at four inbreeding generation intervals, first with the MDA, consisting of 620 000 SNPs,^27^ and later with MUGA, consisting of 7500 markers, and finally with Mega‐MUGA genotype array, consisting of 77 800 markers to confirm their genotype status.^51^

### 2.7 Genotype‐phenotype linkage analysis

Quantitative trait locus mapping was performed using the baseline liver weight phenotypic data and the genotypic data of the CC lines using HAPPY software.^28^ The QTL mapping was performed in three directions, once for the overall mouse population, then separately by sex. The mapping of QTL at SNP interval (*L – locus*) of CC line (*k*) was tested using the linear regression framework below, in which the HMM probability of descent from founder strain (*s*) is denoted by *P*_*LK(s)*_:

$$\ln\frac{\pi (y_{k})}{1-\pi (y_{k})}=\mu +\sum_{s}P_{\mathrm{LK}}\left(s\right)\beta_{s}$$

where *K = *CC line; *L* = locus, *s* = founder strain, *y*_*k*_ = residual deviance from the mean probability of death for an individual line *k*, β_s_ = the effect of founder haplotype (*s*) at locus (*L*), null hypothesis β_s_ = 0.

Significance level presented as the negative log_10_ of the *P*‐value of null hypothesis test (R ANOVA). Estimation of genome wide significance was performed by permutation test, in which CC line labels were permuted between the phenotypes. Further details of the QTL approach used in this study are available in our previous studies.^31^, ^32^, ^33^, ^34^, ^35^, ^37^

## 3 RESULTS

### 3.1 Liver weight

**Figure 1:**
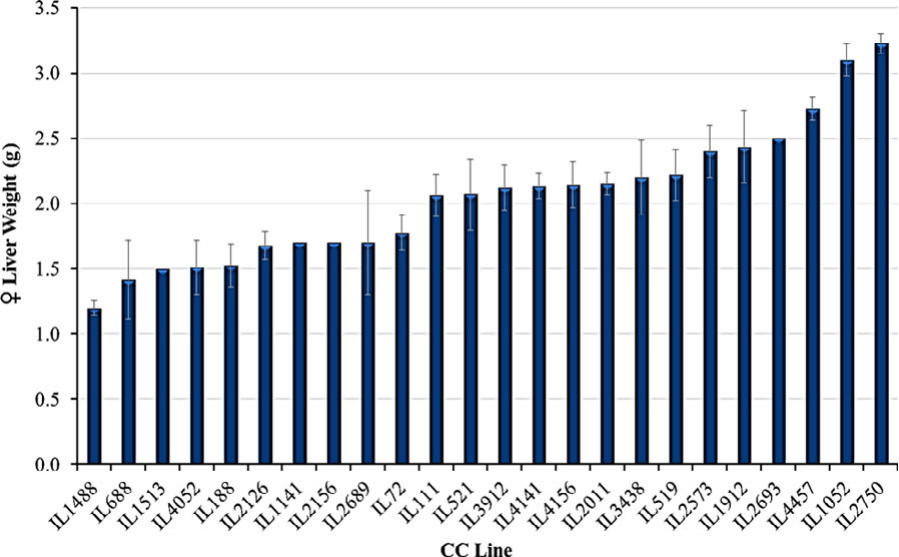
Means of liver weight (g ± standard error of the mean) of female mice from 24 Collaborative Cross (CC) lines measured at 20 weeks of age by manual assessments using an electronic balance. *X*‐axis represents the different CC lines and *Y*‐axis represents liver weight values in grams measured by manual assessments using an electronic balance. Means of liver weight (g) differ significantly between the CC lines (*P* < 0.01)

Our findings demonstrate a significant profile of phenotypic variations between the CC lines for the liver weight, suggesting empirical evidence for the strong genetic component controlling the liver weight. Two‐way anova for sex * line interactions was significant (*P* = 0.01) indicating that females and males differ significantly across CC lines in their liver weight; therefore, we analyzed data separately by sex. Although the tested cohort was kept under controlled, common environmental conditions, one‐way anova for variations of mean liver weight in grams (g) between the CC lines showed highly significant variations for the overall population and also separately for both sexes (overall population *P* = 8.71e^−18^, female mice *P *= 5.65e^−09^, male mice *P *= 2.08e^−15^). For female mice cohort, liver weight ranged 1.20 g (±0.055) to 3.23 g (±0.075), line IL1488 with the lowest value, while line IL2750 had the highest. As for the male mice cohort, liver weight ranged 1.27 g (±0.12) to 4.53 g (±1.00), line IL1468 with the lowest value, while line IL4799 had the highest. The mean liver weight for the overall CC population of the study cohort was 2.17 g (±0.04), while 2.02 g (±0.05) and 2.24 g (±0.05) for females and males, respectively. H^2^ and CV_G_ were calculated for liver weight and found to be, respectively, 0.37 and 0.22 g for females, and 0.33 and 0.24 g for males. Figures 1 and S1 show the means of liver weight for females and males, respectively, of the different CC lines at the age of 20 weeks, following 12 weeks on standard rodent diet and naive environmental conditions.

### 3.2 QTL mapping and founder effect

**Figure 2:**
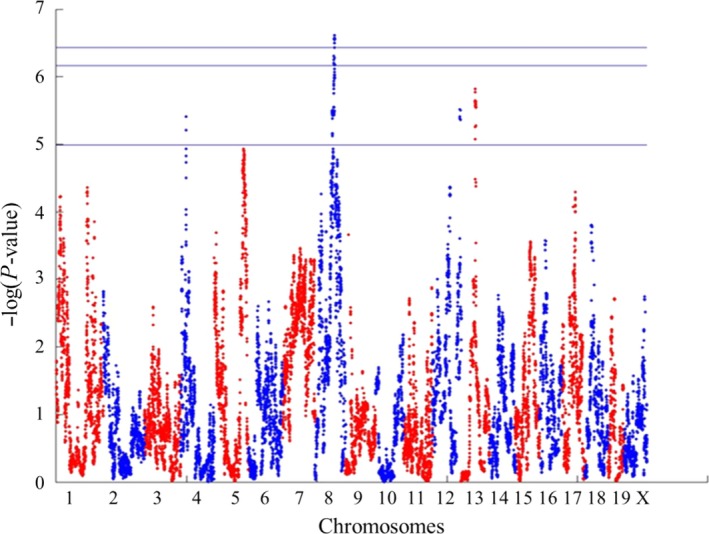
Genomic location of the significant quantitative trait loci (QTL) associated with liver weight (g) of different Collaborative Cross (CC) lines. QTL associated with liver weight trait detected on chromosome 8. Experiment‐wide thresholds of significance at *P% of 50%, 90% and 95% levels are logP = 4.99, 6.16 and 6.43, respectively.*P% threshold means that in P% of permutations the genome‐wide maximum logP across all analyses at different time points did not exceed the threshold

Initially, 1000 randomization tests were performed to calculate the 5%, 10% and 50% genome‐wide significant thresholds. The three thresholds are presented on a Manhattan plot in Figure 2, and found to be logP = 6.43, 6.16 and 4.99 at 5%, 10% and 50% at genome‐wide significance, respectively.

A significant QTL peak at 5% genome‐wide significance was mapped on chromosome 8 at a genomic interval 88.61‐93.38 Mb. This obtained a genomic interval of 4.77 Mb, which is considered a high‐resolution mapping result, knowing that the mapping population consists of only 24 CC lines.

Three additional QTL peaks were mapped at 50% genome‐wide significance threshold on chromosomes 4, 12 and 13 at genomic intervals of 29.39‐30.02 Mb, 115.59‐117.82 Mb and 62.94‐65.15 Mb, respectively. Calculating the mapped genomic intervals of each of these three QTL, we found that the suggested regions are 0.63 Mb, 2.23 Mb and 2.21 Mb for chromosomes 4, 12 and 13 QTL, respectively. Table 1 summarizes the four QTL, positions and intervals.

**Table 1:** Genomic locations of quantitative trait loci (QTL) associated with females’ liver weight (g) trait of 24 Collaborative Cross (CC) lines. Chr, chromosome; logP, negative log_10_ *P*‐value; Sig, genome‐wide significance level reached, genomic position and length of the 50%, 90% and 95% confidence intervals relative to mouse genome build mm9; *LWL*1‐4, liver weight locus 1‐4, names of the mapped QTL

QTL	Chr.	logP	Sig.	50% CI (Mb)		90% CI (Mb)		95% CI (Mb)	
				Position	Width	Position	Width	Position	Width
*LWL1*	8	6.62	0.05	88.61‐93.38	4.77	88.61‐93.38	4.77	88.61‐93.38	4.77
*LWL2*	4	5.33	0.50	29.39‐30.02	0.63	‐	‐	‐	‐
*LWL3*	12	5.51	0.50	115.59‐117.82	2.23	‐	‐	‐	‐
*LWL4*	13	5.78	0.50	62.94‐65.15	2.21	‐	‐	‐	‐

The four QTL were named *LWL*1‐*LWL*4 referring to liver weight locus 1‐4. The QTL on chromosomes 8, 4, 12 and 13 were designates with the names *LWL*1, *LWL*2, *LWL*3 and *LWL*4, respectively.

**Figure 3:**
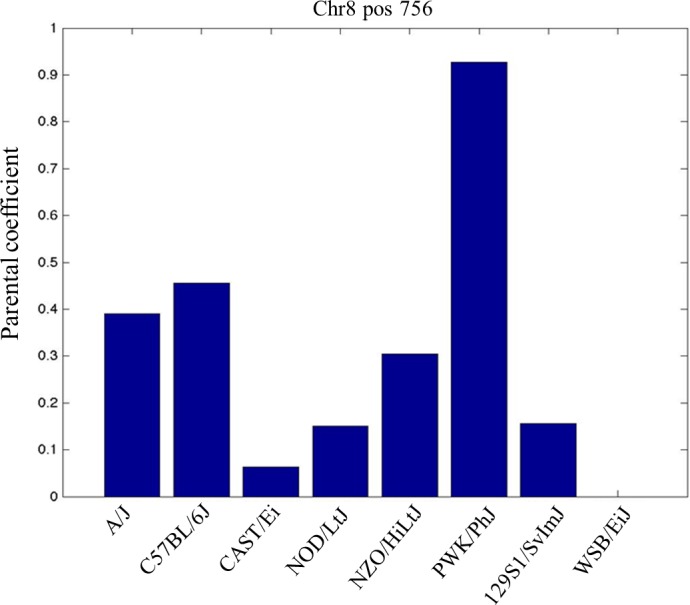
Estimated haplotype effect size at chromosome 8 quantitative trait loci (QTL) for liver weight (g) trait. Effects are shown as deviations relative to WSB/EiJ, which is arbitrarily assigned the trait effect of 0. The *X*‐axis represents eight founder strains of the CC lines; *Y*‐axis represents the estimated haplotype effect size of the CC founders

Finally, the effect of each founder haplotype on liver weight at the QTL on chromosome 8 was calculated as deviation relative to WSB/EiJ, which is arbitrarily assigned the trait effect of 0. Results of this analysis are presented in Figure 3. The locus showed a complex pattern of haplotype effects of the founders, with the wild‐derived strains, mainly PWK, playing a major role but other strains also contributing to the overall QTL effect. QTL analysis was not significant neither for males nor for the overall population.

### 3.3 Candidate genes underlying the mapped QTL

The mapped QTL genomic intervals were searched using the mouse genome database (http://www.informatics.jax.org/) for identifying the highly suggested candidate genes that underlined these QTL.

Indeed, the search results revealed several genes and overlapping QTL that were involved in various physiological processes, including cellular pathways for energy metabolism, inflammatory pathways, fatty acids metabolism at different levels, growth and bodyweight regulation, and insulin‐mediated glucose regulation.

We focused our candidate gene analysis on the significant QTL at chromosome 8, *LWL*1. A gene browser search within the *LWL*1 genomic interval revealed 155 features, from which 28 features are protein coding genes and 11 overlapping QTL. Focusing on the suggested protein coding genes, we observed a cluster of the mouse carboxylesterase 1 (*Ces1*) family, consisting of eight genes, *Ces1a‐Ces1h* (MGI: 3648919, 3779470, 95420, 2148202, 95432, 2142687, 88378 and 1922954, respectively) comprising the hepatic carboxylesterase family proteins playing major roles in lipid metabolism and xenobiotic clearance.^13^, ^53^ Increased attention to the carboxylesterase family is due to potential roles as cholesterylester and/or triacylglyceride hydrolases; so far, *Ces1d* has been identified as a triglyceride hydrolase^54^ which is regulated by inflammatory factors (interleukin‐6, transforming growth factor‐β and tumor necrosis factor‐α).^55^ A recent study of the role of *Ces*1d in hepatic liver metabolism shows that *Ces*1d deficiency in mice ameliorates the hepatic steatosis, suggesting possible potential in non‐alcoholic fatty liver disease (NAFLD) therapy.^56^ The co‐location of the *Ces*1 family cluster within the *LWL*1 QTL for liver size suggests sharing common expression regulators, responding to liver growth also at the level of metabolic function to address the body metabolic demands. Another organized gene cluster with high relevance to growth and development^57^, ^58^ was observed within the *LWL*1 QTL, the Iroquois homeobox genes complex B (*IrxB*), consisting of the three genes *Irx*3, *Irx*5 and *Irx*6 (MGI: 1197522, 1859086 and 1927642, respectively). The presence of the *Irx*B cluster induces the complexity of the *LWL*1 region due to their multiple regulatory roles in early development. Adjacent to the *Irx*B cluster, within the significant *LWL*1 QTL, we located the fat mass and obesity‐associated (*FTO*) gene and Retinitis pigmentosa GTPase regulator interacting protein 1‐like (*Rpgrip*1l) gene, together identified as a topologically associated domain (TAD) structure in which any transcriptional perturbation during development may potentially affect bodyweight index.^59^ The *Rpgrip*1l gene (MGI: 1920563) is highly involved in growth/early development and suggested to be a tumor suppressor of hepatocellular carcinoma.^60^

The *FTO* gene (MGI: 1347093), encoding the fat mass and obesity‐associated protein, was the first GWAS‐identified obesity and obesity‐related trait‐associated gene (ie, type 2 diabetes, hip circumference, bodyweight index, bodyweight).^61^, ^62^ Owing to the various studies of *FTO* association with obesity and obesity‐related phenotypes, it is now evident that *FTO* is highly expressed in adipose tissues, playing a crucial role in adipogenesis (cross‐talk with *Irx3*) and extremely involved in early development, and yet its function is still obscure beyond the adipose tissue.^63^ In relevance to liver weight phenotype, studies of *FTO* homozygous, null mice reported postnatal growth retardation accompanied by decreased bodyweight (lean and fat weight), suggesting the complex and major role of *FTO* in body development and composition, whether independently or by co‐regulatory mechanisms with the *Irx*B cluster and *Rpgrip*1l gene.^64^ Hence, perturbation of the transcriptional architecture within this region during development could potentially affect any or all of these genes, and lead to altered growth phenotypes and tumors. Several studies revealed *FTO* gene association with NAFLD, colorectal cancer and pancreatic cancer to be further examined.^65^ Another candidate gene located within the *LWL*1 interval, named the Retinoblastoma‐like 2 gene (*Rbl*2; MGI: 105085), member of the Retinoblastoma (Rb)/Rb‐like protein family, plays an important role in cell differentiation and early development, and therefore suggested to be involved in growth interruption and tumors.^66^, ^67^ The *Rb* gene family has been reported to be involved in regulatory pathways with organ size control mechanisms of the body, inactivation of the Rb pathway using Rb family knockout mouse model, resulting in development of liver tumors, implying its strong regulatory role in control of cell proliferation.^68^, ^69^ One more candidate gene, matrix metallopeptidase 2 (*Mmp*2; MGI: 97009), belonging to the matrix metalloproteinase (MMP) family. *Mmp*2 plays an essential role in tissue growth at the regulatory level of angiogenesis, proteolytic remodeling processes of the extracellular matrix and adipogenesis during growth.^70^ *Mmp*2 null mutation mice show a development delay and reduced body size compared with wild types, as well as fat malformation at the adipose tissue suggesting an important role in adipogenesis.^71^, ^72^ Additionally, the Calpain, small subunit 2 *Capns*2 gene (MGI: 1916793), member of the Calpin system, plays a crucial role in regulation of angiogenesis, development and cancer, reported to be in association with various physiological and pathological processes (eg, type 2 diabetes, cancers, cataract, muscle dystrophy).^73^

Further genes included within the *LWL*1 genomic interval are found to be related to growth, development, body size, metabolism and homeostasis such as Solute carrier family 6 (neurotransmitter transporter, noradrenalin), member 2 (*Slc6a*2; MGI: 1270850), CYLD lysine 63 deubiquitinase (*Cyld*; MGI: 1921506), Thymoma viral proto‐oncogene 1 interacting protein (*Aktip*; MGI: 3693832), Nucleotide‐binding oligomerization domain containing 2 (*Nod*2; MGI: 2429397) and TOX high‐mobility group box family member 3 (*Tox*3; MGI: 3039593).

Interestingly, 11 previously reported QTL were overlapped with *LWL*1 genomic interval, including two QTL related to body growth, namely obesity phenotype, called Obesity QTL 16 (*Obq*16), and bodyweight, called Weight 3 weeks QTL 5 (*W3q*5). Another two QTL were associated with immune response phenotypes, named *Plasmodium chabaudi* malaria resistance QTL 2a (*Char*2a) and experimental allergic encephalomyelitis susceptibility 31 (Eae31).

Beside protein coding genes and QTL, abundant 5′‐C‐phosphate‐G‐3′ islands (CpG islands) (39 features) were located within the significant genomic interval of *LWL*1, which is known to be associated with promoters of housekeeping genes and genes with a tissue‐restricted pattern of expression.^74^ CpG island distribution varies within the whole genome to be in preference for gene‐rich loci; advanced studies of epigenetics and DNA methylation propose a possible important role of CGI methylation in mammalian development and cellular differentiation.^75^ Additionally, the *LWL*1 genomic intervals contains 25 long non‐coding RNA (lncRNA) genes (encoding a non‐coding RNA, length >200 nucleotides), seven large intervening non‐coding RNA (lincRNA) genes and three miRNA genes.

## 4 DISCUSSION

To our knowledge, herein, we present, for the first time precise mapping of sex‐specific QTL influencing the baseline liver weight phenotypic variations of female mice generated from 24 CC lines. Considering the accumulated evidence for significant sex differences at baseline traits/disease etiology, and based on our previous studies where we demonstrated the significant sex differences observed between sexes within the CC lines,^34^, ^35^, ^76^ we have therefore included both sexes in our current analysis. Nevertheless, mapped QTL were significant only for females’ data, while neither males nor overall population showed significant QTL.

In the current study, we introduce a novel sex‐specific QTL associated with basal female mice liver weight (g). Dissecting the genetic components controlling baseline liver weight will enhance the understating of medical conditions of growth retardation and diseases involving hepatomegaly, whether as major symptoms or secondary.

With joint community efforts, the CC mouse model was generated to deal with the necessity for a new model population designated as a highly genetically diverse model improving the QTL mapping resolution and accordingly contributing to the understanding of complex trait/etiologies of human diseases.^22^, ^23^ Consequently, the CC inbred lines were created from full reciprocal matings of eight parental mouse strains; five known laboratory models (A/J, C57BL/6J, 129S1/SvImJ, NOD/LtJ, NZO/HiLtJ) and three wild derivatives (CAST/Ei, PWK/PhJ and WSB/EiJ) enriching the genetic architectures of the new inbred lines. Persistent inbreeding of the CC lines is ongoing for generations at the small animal facility of TAU.

The significant QTL, named *LWL*1 (liver weight locus 1), is located on chromosome 8 at genomic interval 88.61‐93.38 Mb (4.77 Mb), which is to our knowledge the narrowest reported QTL thus far for baseline liver weight trait in mice. Furthermore, 24 QTL underlying liver weight were mapped and located at multiple genomic location of different chromosomes emphasizing the genetic complexity of the liver weight phenotypic trait, to be controlled by multiple loci genome wide. Up to this report, the known QTL mapped for liver weight were located at chromosomes 1, 2, 3, 4, 5, 7, 9, 10, 11, 12, 15, X and a suggestive QTL on chromosome 8 for liver weight (see Table S1).^14^, ^77^, ^78^, ^79^, ^80^, ^81^ Certainly, the accumulated data of QTL mapping has expanded the knowledge on genetic architectures controlling liver weight; however, the accurate genetic components are still obscure due to wide genomic intervals (ranging 24.7‐105.8 Mb) leading to a serious impediment for selection of genes of high candidacy. As expected, using the CC mouse population in the current study allowed mapping a significant QTL with exceptional high resolution of 4.77 Mb, thus narrowing the possibilities of candidate genes, bringing us closer to the actual candidate genes controlling liver weight. Indeed, the search for candidate genes within the *LWL*1 significant interval revealed very interesting genes and gene complexes that are known to be associated with body growth and size whether independently or interacting with other genes. Suggested candidate genes of the current study included the *FTO* gene, which is known to be associated with obesity (growth control) and thereafter with tumorigenesis (interrupted growth control), as well as the *Ces*1a to *Ces*1h family, which is related to lipid metabolism and hepatic steatosis, additional to further genes involved in cell proliferation and growth such as *Capns2*,* Mmp2* and *Rbl2*. Moreover, the presence of *IrxB* within the *LWL*1 genomic interval emphasizes that the complexity of liver weight trait may be controlled by and involved in multiple regulatory pathways with cross‐talks between them.

Chromosome 8 was reported previous to its association with growth, development, metabolism and tumorigenesis traits, while 11 QTL were reported overlapping the *LWL*1 genomic interval. Two of the QTL were related to bodyweight/obesity and another two were related to immune response.

The calculated heritability of liver weight trait (*H* ^2^ = 0.37) is considered high confirming the strong genetic components controlling liver growth, enabling fine mapping with high resolution of the QTL with limited number of CC lines (24 CC lines). Furthermore, the calculated value of the genetic coefficient of variation (CV_G_ = 0.22) proves the high genetic variations in liver weight between the CC lines, which is believed to be introduced mainly by the three wild‐derived strains (CAST/Ei (*M. m. castaneus*), PWK/PhJ (*M. m. musculus*) and WSB/EiJ (*M. m. domesticus*).^23^

This report and previous publications^30^, ^31^, ^32^, ^33^, ^34^, ^35^, ^36^, ^37^ demonstrate the utility of the CC mouse model in dissecting complex traits at baseline condition and for complex diseases with complex etiology. Based on founder effect analysis, we show that chromosome 8 QTL, mainly *LWL*1, contributed to by the wild‐derived strain PWK, suggests the possibility of discovering new candidate genes due to the enrichment of the known genetic architectures of laboratory mouse strains by addition of wild‐derived strains.

## CONFLICTS OF INTEREST

None.

## AUTHOR CONTRIBUTIONS

HJA participated in the design of the study, carried out the mice assessment, participated in data analysis and drafting the manuscript. MB participated in the data analyses and edits of the first draft through to the final submitted version. RM participated in designing the study. IGV participated in the data analyses and edits of the first draft through to the final submitted version. FAI participated in designing the study and in developing the first draft through to the final submitted version of the manuscript. All authors read and approved the final manuscript.
