# Supplementary material for: Mapping novel genetic loci associated with female liver weight variations using Collaborative Cross mice
Source: Animal Model Exp Med. 2018 Oct 24;1(3):212–20. doi: 10.1002/ame2.12036 (PMC6388055; doi:10.1002/ame2.12036)
Supplement: Supplementary file 1 [file AME2-1-212-s001.docx]

**Supplementary table 1:**

List of the potential candidate genes located within the *Liver Weight Locus 1*(LWL1) significant QTL and their potential pathways. The candidate genes located within the genomic interval 4.77 Mb of the LWL1 significant QTL, mapped between 88.61-93.38 Mb on Chr8, in linkage with the phenotypic trait of the liver weight of female mice from 24 CC lines. Genes search was performed using the Mouse Genome Informatics (MGI) browser (<http://www.informatics.jax.org/>), and genes potential pathways search in the literature.

| **Candidate gene / gene-cluster** | **Symbol** | **Potential pathways** |
| --- | --- | --- |
| Mouse Carboxylesterase 1 *(Ces1*) family | *Ces1a* to *Ces1h* | - Lipid metabolism and xenobiotic clearance^13, 53^ - Cholesteryl ester and/or triacylglyceride hydrolases regulated by inflammatory factors ^54, 55^ - Hepatic liver metabolism^56^ |
| Iroquois homeobox genes complexB (*IrxB*) | *Irx*3, *Irx*5 and *Irx*6 | - Regulation of growth and development ^57,58^ |
| Fat mass and obesity-associated gene | *FTO* | - Body weight index^59^ - Obesity and obesity related traits associated gene (type 2 diabetes/ hip circumference/ body weight index/ body weight)^61,62^ - Adipogenesis (cross talk with *Irx3*) and early development^63^ - Suggested association with NAFLD, colorectal cancer and pancreatic cancer^65^ |
| Retinitis pigmentosaGTPase regulator interacting protein 1 – like gene | *Rpgrip*1l | - Growth/early development, and body weight index^59^ - Suggested tumor suppressor of the Hepatocellular Carcinoma (HCC)^60^ |
| Retinoblastoma-like 2 gene (Retinoblastoma (Rb)/ Rb-like proteins family) | *Rbl*2 | - Cell differentiation and early development, and suggested to be involved in growth interruption and tumors ^66,67^ - Regulatory pathways of organ size control mechanisms of the body, and control of cell proliferation^68,69^ |
| Matrix metallopeptidase 2  (Matrix Metalloproteinase (MMP) family) | *Mmp*2 | - Tissue growth at the regulatory level of angiogenesis, proteolytic remodeling processes of the Extracellular Matrix (ECM) and adipogenesis during growth^70,71,72^ |
| Calpain, small subunit 2  (Calpin system) | *Capns*2 | - Regulation of angiogenesis, development and cancer, reported to be in association with various physiological and pathological processes (type 2 diabetes, cancers, cataract, muscle dystrophy)^73^ |
| Solute carrier family 6 (neurotransmitter transporter, noradrenalin), member 2 | *Slc6a*2 | - Growth, development, body size, metabolism and homeostasis. |
| CYLD lysine 63 deubiquitinase | *Cyld* |  |
| Thymomaviral proto-oncogene 1 interacting protein | *Aktip* |  |
| Nucleotide-binding oligomerization domain containing 2 | *Nod*2 |  |
| TOX high mobility group box family member 3 | *Tox*3 |  |
