# Supplementary figures and images for: Mapping novel genetic loci associated with female liver weight variations using Collaborative Cross mice
Source: Animal Model Exp Med. 2018 Oct 24;1(3):212–20. doi: 10.1002/ame2.12036 (PMC6388055; doi:10.1002/ame2.12036)

**Table 1 Supplement.**


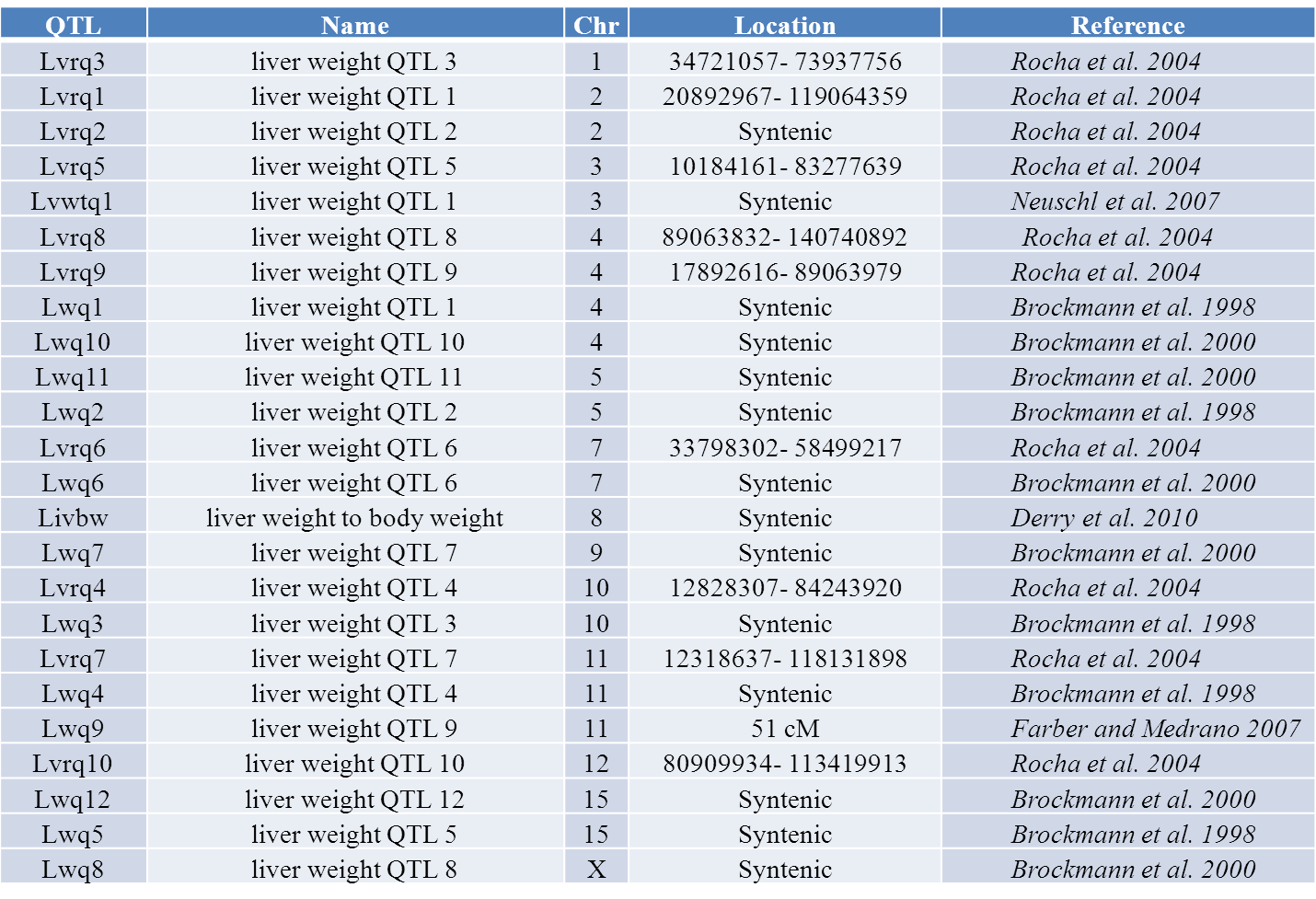


**Figure 1 Supplement.**


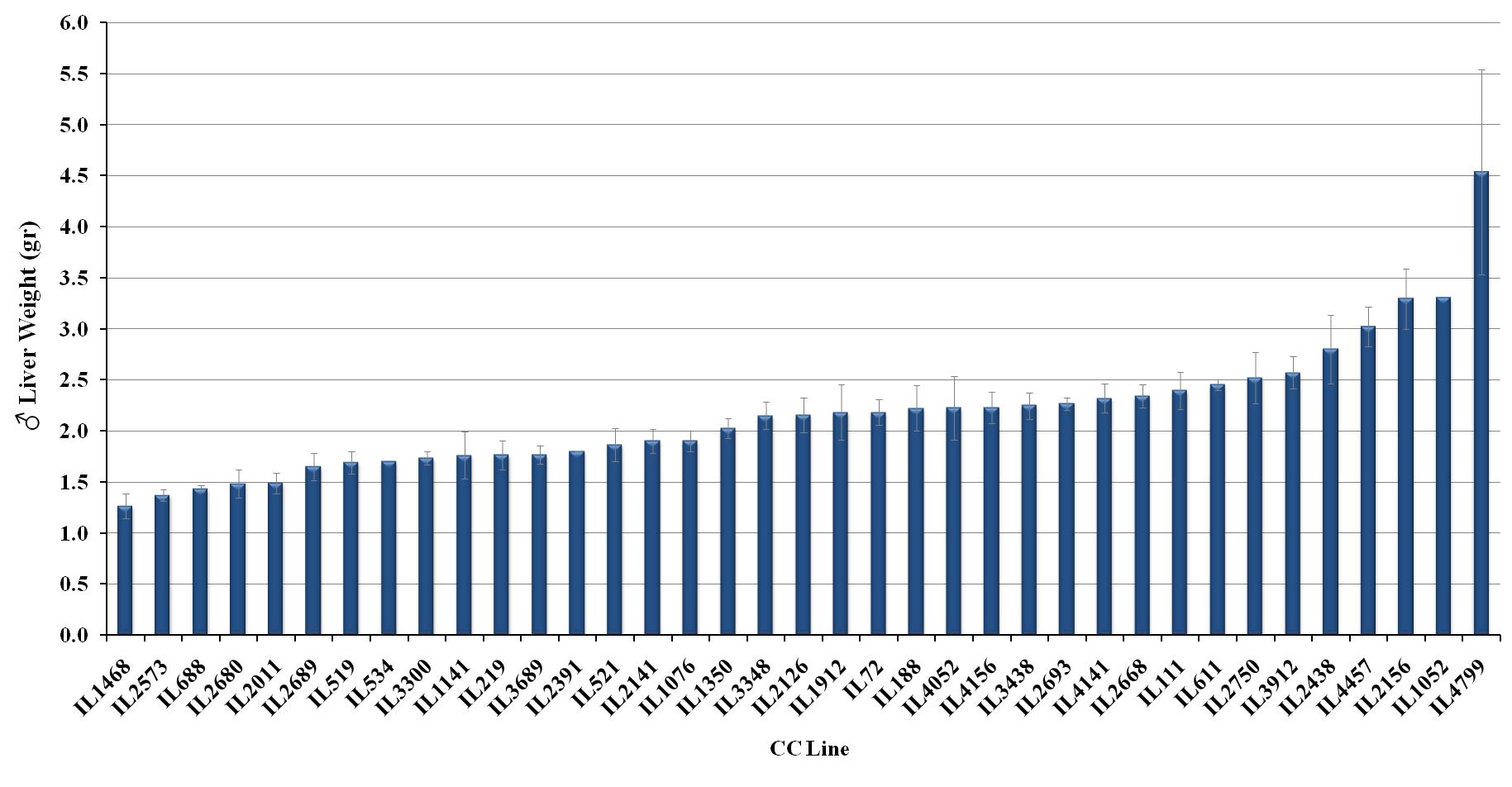

Supplement: Supplementary file 2 [file AME2-1-212-s002.docx]
